# Pelvic Fractures in Pregnancy: Multidisciplinary Management and Outcomes

**DOI:** 10.7759/cureus.84898

**Published:** 2025-05-27

**Authors:** Kaan Sevgi, Steven Pliskow, Sinan Nazif Aran

**Affiliations:** 1 School of Medicine, Kansas City University of Medicine and Biosciences, Kansas City, USA; 2 Department of Obstetrics and Gynecology, HCA Florida Palms West Hospital, West Palm Beach, USA; 3 Department of Family Medicine, Demiroglu Science University, Istanbul, TUR

**Keywords:** fetal health, maternal health, multidisciplinary management, pelvic fractures, pregnancy, trauma

## Abstract

Pelvic fractures during pregnancy, though rare, pose significant risks to maternal and fetal health, necessitating a multidisciplinary approach to management. These injuries are predominantly caused by high-energy trauma, such as motor vehicle accidents, and are associated with a high incidence of complications, including maternal hemorrhage, placental abruption, and preterm labor. Advances in diagnostic imaging, such as low-dose CT scans and MRIs, have improved diagnostic accuracy while minimizing fetal exposure to radiation. Similarly, biomechanically tailored surgical interventions and innovative treatment strategies, including minimally invasive fixation techniques, have enhanced maternal survival rates, reaching 95% when multidisciplinary care is implemented. Despite these advancements, challenges persist, particularly in resource-limited settings where disparities in access to care result in poorer outcomes. Ethical and medico-legal considerations further complicate management, emphasizing the importance of informed consent and balancing maternal autonomy with fetal well-being. This review highlights the critical role of public health initiatives, such as trauma prevention campaigns, and the need for innovation in diagnostics, therapeutics, and long-term care. Future research should prioritize addressing gaps in our understanding of long-term maternal and neonatal outcomes, particularly regarding reproductive health and quality of life. By integrating advanced technologies, fostering collaboration, and improving global access to care, clinicians can achieve optimal outcomes for both mother and child.

## Introduction and background

Pelvic fractures during pregnancy, while rare with an estimated incidence of 1 in 30,000 pregnancies, present a formidable clinical challenge due to the need to manage both maternal and fetal health concurrently. These injuries are most often the result of high-energy trauma, particularly motor vehicle accidents, and carry a high risk of adverse outcomes, including hemorrhage, preterm labor, and fetal demise [1]. Trauma-induced physiological stress can complicate the maternal hemodynamic response, making early stabilization and monitoring essential. Although anticoagulation for venous thromboembolism prophylaxis is frequently considered in trauma settings, its safety in pregnant trauma patients, especially those with coexisting intracranial injuries, must be carefully weighed [1].

The anatomical and physiological changes that occur during pregnancy, such as increased pelvic vascularity, joint laxity, and displacement of pelvic structures by the enlarging uterus, heighten the complexity of diagnosis and treatment [2]. These adaptations increase the vulnerability of the pelvic ring to injury under mechanical stress and make interpretation of imaging and symptoms more difficult. For example, peripartum pubic symphysis diastasis may mimic or accompany traumatic fractures, presenting with severe pelvic pain and gait disturbances [2]. Moreover, these conditions can lead to prolonged disability if not promptly diagnosed and appropriately managed.

Imaging is fundamental to management, but clinicians must balance diagnostic clarity with fetal safety. MRI and ultrasound are preferred during pregnancy due to their safety profiles, whereas low-dose CT and X-rays may be used when critical clinical information is needed [3]. In cases of unstable fractures, surgical management is often necessary and must be carefully tailored. Modifications in patient positioning and anesthetic technique, such as using the left lateral tilt to prevent aortocaval compression, are essential to ensure maternal and fetal well-being [3]. Operative planning must also consider the timing of the procedure relative to gestational age and fetal viability.

Treatment strategies vary depending on fracture type, stability, and gestational age. Stable fractures may be treated conservatively with pelvic binders, bed rest, and activity restriction. In contrast, unstable or displaced fractures often require internal fixation to restore anatomical alignment and allow early mobilization [4]. Advances in minimally invasive surgical techniques and the use of external fixators have contributed to better maternal outcomes and reduced operative morbidity [4]. However, surgical intervention must be weighed against the risks of anesthesia, blood loss, and preterm labor.

Despite these advances, standardized guidelines for managing pelvic fractures in pregnant patients remain limited. Most treatment decisions rely on case series, expert opinion, or protocols extrapolated from non-pregnant populations. Publications such as those from Tejwani et al. underscore the need for comprehensive, interdisciplinary care pathways that incorporate obstetricians, trauma surgeons, anesthesiologists, and neonatologists to optimize outcomes [5]. Such collaboration is especially important in centers without dedicated maternal trauma units.

Beyond clinical management, prevention remains a critical but underemphasized strategy. Public health initiatives targeting women of childbearing age, such as seatbelt safety campaigns, fall prevention, and intimate partner violence screening, can substantially reduce the incidence of trauma during pregnancy. Cannada and Barr highlight that young, active women are particularly at risk for high-energy pelvic trauma and that these events can have long-term implications for mobility, fertility, and psychological well-being [6]. Therefore, pelvic fractures in pregnancy should be viewed not only as an orthopedic emergency but as a multidisciplinary care priority with long-term health implications for both mother and child.

This review synthesizes current knowledge on the diagnosis, management, and prevention of pelvic fractures in pregnancy. By emphasizing early diagnosis, interdisciplinary collaboration, evidence-based decision-making, and preventive care, it aims to equip clinicians with the tools needed to improve maternal and fetal outcomes in this rare but high-stakes clinical scenario.

## Review

Pelvic fractures during pregnancy represent a complex medical challenge with significant implications for maternal and fetal health. Although rare, occurring in approximately 1 in 25,000 to 30,000 pregnancies, these injuries can have devastating consequences for both mother and child [1]. The rarity of these fractures limits clinician experience, making evidence-based guidance essential. High-energy trauma, such as motor vehicle accidents, accounts for up to 60% of cases, while falls (20%) and direct trauma (15%) comprise the remaining causes [2]. Physiological changes in pregnancy, including increased pelvic mobility and ligament laxity, further complicate the management of these injuries [3].

During pregnancy, the body undergoes significant anatomical and physiological adaptations to accommodate the growing fetus and prepare for childbirth. These include increased blood volume, altered hemodynamics, and musculoskeletal changes such as ligament relaxation and pelvic widening. While these changes are beneficial for childbirth, they heighten the risks associated with trauma. For instance, increased vascularity in the pelvic region during pregnancy significantly raises the risk of life-threatening hemorrhage in cases of pelvic fractures [4].

This review synthesizes the current evidence on the epidemiology, pathophysiology, diagnosis, management, and outcomes of pelvic fractures in pregnancy. The goal is to provide clinicians with a comprehensive, data-driven framework to guide decision-making while addressing existing gaps in the literature. Multidisciplinary collaboration, integrating expertise from orthopedic surgeons, obstetricians, anesthesiologists, and critical care specialists, is essential to achieve optimal outcomes in these high-risk cases [5]. Additionally, public health measures, such as improved road safety initiatives and fall prevention education for pregnant women, hold the potential to reduce the incidence of trauma-related pelvic fractures [6].

Despite advancements, managing pelvic fractures in pregnancy remains among the most demanding challenges in medical practice, as it requires balancing maternal and fetal health outcomes. This review highlights actionable insights, evidence-based strategies, and opportunities for future research to enhance care for this vulnerable population. By bridging gaps in knowledge, this review aims to promote innovation and elevate the standard of care for pregnant patients with pelvic trauma.

Epidemiology and risk factors

Pelvic fractures in pregnancy, though infrequent, are disproportionately associated with adverse outcomes. High-energy trauma remains the primary cause, with motor vehicle accidents contributing to 60% of cases, falls to 20%, and direct trauma to 15% [2]. The global distribution of these injuries reflects variations in road safety, access to prenatal care, and socioeconomic factors. For instance, low-income regions often report higher incidences of trauma-related fractures due to inadequate road infrastructure and limited enforcement of traffic laws [6]. Maternal mortality rates in pelvic fractures during pregnancy range from 10% to 15%, with the most severe injuries associated with unstable fractures and massive hemorrhage [3,4]. Fetal outcomes are similarly concerning, with increased risks of placental abruption (20%) and preterm labor (30-50%) in cases of severe maternal trauma [5]. The physiological changes of pregnancy, including increased vascularity and ligamentous laxity, exacerbate these risks [3]. Furthermore, the compression of abdominal organs and displacement of the uterus in late pregnancy can alter the presentation and severity of trauma, complicating diagnosis and management. In multi-gestational pregnancies or in the presence of comorbidities such as preeclampsia, the risk profile intensifies, making even minor trauma clinically significant. Risk factors for pelvic fractures extend beyond trauma. Conditions such as osteoporosis, often exacerbated by pregnancy-induced calcium demands, increase the susceptibility of the pelvis to fractures even with low-energy mechanisms. Additionally, ligamentous laxity, a normal adaptation to facilitate delivery, predisposes the pelvis to instability under mechanical stress [3]. The increasing maternal age in many populations further contributes to the prevalence of osteoporosis-related fragility fractures. Preventive measures play a crucial role in reducing the incidence of pelvic fractures. Prenatal education programs that address fall prevention and road safety for pregnant women can mitigate the risk of trauma. Similarly, public health campaigns focusing on seatbelt use and safe driving practices can reduce the incidence of motor vehicle accidents, the leading cause of these injuries [6]. Occupational safety guidelines for pregnant women in physically demanding roles also warrant consideration.

Despite the availability of data on risk factors and epidemiology, there is a need for more region-specific research to tailor prevention and management strategies. Geographic disparities in the burden of trauma-related pelvic fractures highlight the importance of localized public health interventions. Future studies should focus on identifying modifiable risk factors and evaluating the effectiveness of preventive measures, such as prenatal education programs and legislative changes.

Pathophysiology and pregnancy-specific considerations

Pregnancy induces profound anatomical and physiological adaptations that significantly alter the biomechanics of the pelvis and increase vulnerability to injury. Hormonal fluctuations, particularly elevated levels of relaxin and progesterone, lead to marked ligamentous laxity, facilitating the softening of connective tissues and expansion of the pelvic ring to accommodate childbirth. This hormonally mediated change results in a 20-30% increase in pelvic mobility, which, although functionally necessary for vaginal delivery, compromises the structural stability of the pelvic girdle and heightens the risk of injury even with minor trauma [3]. Concurrently, the maternal pelvis is subjected to increased biomechanical stress due to an approximate 30% rise in weight-bearing load during pregnancy, compounding the risk of fracture in the presence of instability [7].

Hemodynamically, the pregnant body undergoes a 40-50% increase in blood volume and cardiac output to support the growing fetus. This hyperdynamic circulatory state serves as a buffer against hemorrhagic events, offering some physiological reserve. However, it simultaneously poses a heightened risk during trauma, particularly in the case of unstable pelvic fractures, where pelvic vascular disruption can lead to catastrophic hemorrhage and is a leading cause of maternal mortality [8,9]. The engorgement of pelvic vasculature during pregnancy further amplifies the risk of uncontrolled bleeding in such scenarios.

Fetal well-being is inextricably tied to maternal hemodynamic status. Trauma-induced hypotension or shock can reduce uteroplacental perfusion, leading to complications such as intrauterine growth restriction, fetal distress, or stillbirth. Direct trauma to the gravid uterus or placental separation, reported in 20% of pelvic fracture cases, can initiate preterm labor, which occurs in 30-50% of such incidents [5,8].

The unique biomechanical and vascular changes of pregnancy necessitate the development of tailored orthopedic interventions. Current fixation systems and surgical methods are primarily designed for non-pregnant anatomy, often overlooking the altered joint laxity and dynamic load-bearing capacity of the gravid pelvis. Future innovations should prioritize flexible yet stable fixation strategies and minimally invasive approaches that accommodate both anatomical changes and fetal safety.

Diagnosis

Accurate and timely diagnosis of pelvic fractures in pregnant patients is critical for optimizing maternal and fetal outcomes, yet it remains one of the most challenging aspects of trauma care during pregnancy. The diagnostic dilemma arises from the need to comprehensively assess maternal injuries while simultaneously minimizing fetal exposure to ionizing radiation. A strategic balance must be maintained between clinical urgency and imaging safety, often requiring a tailored approach based on gestational age, trauma severity, and resource availability.

Low-dose X-rays are typically the first-line imaging modality due to their accessibility and speed. Advances in digital radiography and shielding techniques have significantly reduced fetal radiation exposure, rendering them relatively safe when judiciously applied, especially during the second and third trimesters [10]. However, X-rays may fail to reveal subtle or complex disruptions in the pelvic ring, prompting the need for more advanced imaging.

CT scans, offering superior spatial resolution and detailed visualization of bone architecture, are invaluable for assessing fracture severity and planning surgical interventions. While concerns about radiation persist, recent studies have demonstrated that pelvic CT scans can be safely performed in pregnant women using abdominal shielding, particularly when maternal stabilization is at stake [11]. The benefits often outweigh the theoretical risks in life-threatening situations.

MRI is the gold standard for evaluating soft tissue injuries, including ligamentous damage and retroperitoneal hematomas, without exposing the fetus to radiation. Despite its diagnostic superiority, MRI’s limited availability and high cost can restrict its use, especially in emergency or low-resource settings [10]. In such scenarios, portable ultrasound plays a crucial adjunct role, not for assessing the fracture itself but for evaluating fetal well-being and complications such as placental abruption and intrauterine bleeding [11].

Clinical evaluation must accompany all imaging decisions. Physical signs such as pelvic instability, disproportionate pain, and hemodynamic changes should prompt immediate diagnostic workup. Fetal assessment through non-stress testing and biophysical profiling is equally vital, as maternal trauma can lead to acute fetal distress [10].

The absence of standardized diagnostic algorithms for pregnant trauma patients delays recognition and intervention. Implementing universal protocols for imaging and clinical triage, stratified by trimester and injury severity, would significantly enhance diagnostic precision. Future innovations in portable and low-radiation imaging technologies could revolutionize point-of-care diagnostics, especially in austere or prehospital environments.

Management approaches

The management of pelvic fractures in pregnancy demands a nuanced and multidisciplinary strategy that ensures maternal stabilization while prioritizing fetal safety. Given the dual physiological considerations, every intervention must be weighed for both immediate and long-term consequences. Management strategies are generally categorized into non-surgical and surgical modalities, selected based on the type and severity of the fracture, gestational age, maternal hemodynamic stability, and the presence of any obstetric complications.

Multidisciplinary Approach

Effective management of pelvic fractures in pregnancy begins with early activation of a multidisciplinary team, whose roles are not only complementary but interdependent. Orthopedic surgeons assess fracture morphology and determine the need for conservative versus surgical treatment, often making decisions in tandem with obstetricians who evaluate gestational age, placental positioning, and signs of fetal distress. Anesthesiologists face the complex task of selecting anesthesia protocols that ensure maternal cardiovascular stability while avoiding agents that could affect uterine tone or fetal oxygenation. In more complex cases, maternal-fetal medicine specialists, neonatologists, and critical care physicians must be engaged early. The inclusion of social workers and psychological support services is also valuable, given the emotional toll such trauma imposes on expectant mothers. Moreover, effective communication and a shared decision-making framework involving the patient and her family are central to achieving holistic care. Regular case conferences and real-time decision-making protocols can facilitate rapid responses to evolving clinical conditions. As trauma systems become increasingly regionalized, the development of specialized trauma centers with obstetric capabilities could significantly improve maternal-fetal outcomes in such high-stakes cases [12,13].

Non-surgical Management

Non-surgical management of pelvic fractures in pregnancy requires a meticulous and individualized care plan tailored to both maternal status and fetal maturity. The use of pelvic binders and external stabilization devices must be applied with caution to avoid excessive uterine compression, which can impair placental perfusion. Pain control typically involves a multimodal regimen, including acetaminophen, short-course opioids, and, in some cases, epidural analgesia, which can simultaneously manage pain and facilitate early ambulation. Bed rest, although necessary for stabilization, is a double-edged sword; it increases risks of thromboembolic events, deconditioning, and urinary complications. Therefore, prophylactic anticoagulation with low-molecular-weight heparin is often considered unless contraindicated [14,15]. Frequent reassessment is vital; deterioration in maternal hemodynamics, increasing pelvic instability, or signs of fetal distress may necessitate a shift to surgical management. Additionally, in late pregnancy, monitoring fetal heart rate patterns and uterine activity becomes crucial to detect complications such as placental abruption or preterm labor. Physical therapy should be integrated as soon as the patient is stable to minimize functional loss and prepare for eventual mobilization and childbirth. Post-discharge planning should also include strategies to prevent falls and ensure continuity of prenatal care.

Surgical Management

Surgical management of pelvic fractures in pregnancy presents unique technical and ethical challenges. Surgical timing must carefully consider maternal urgency versus fetal viability, particularly in the second and third trimesters. Procedures are often performed under general anesthesia with uterine shielding and fetal monitoring when feasible. The prone or supine position must be adjusted to prevent aortocaval compression, often requiring lateral tilt positioning. External fixation remains the preferred initial technique due to its ability to quickly stabilize the pelvis with minimal intraoperative exposure. In some cases, staged fixation strategies are adopted: initial external fixation followed by definitive internal fixation postpartum. Emerging technologies such as intraoperative three-dimensional navigation, low-dose fluoroscopy, and augmented reality systems are being explored to improve surgical precision while safeguarding the fetus [16]. Despite these innovations, the procedure still carries inherent risks, including infection, thromboembolism, and preterm labor. Postoperative care demands close monitoring for uterine contractions, hemorrhage, and deep vein thrombosis. Additionally, obstetric plans may need to be revised based on post-fracture pelvic anatomy, sometimes necessitating cesarean delivery if anatomical distortions impede vaginal birth. Long-term follow-up is essential to assess functional outcomes, pelvic alignment, and psychological well-being. Surgical teams must also consider implant biocompatibility in future pregnancies, especially when internal fixation hardware remains in place.

Despite advancements in surgical safety and imaging, significant gaps remain in our understanding of the long-term effects of surgical interventions on reproductive outcomes. There is limited data on future pelvic function, mode of delivery in subsequent pregnancies, and the impact of implants on pelvic biomechanics during labor. Moreover, resource-limited settings often lack access to specialized trauma and obstetric care, which can delay definitive treatment and worsen outcomes. Developing scalable, cost-effective treatment algorithms, including mobile trauma teams, telemedicine consultations, and modular surgical kits, could bridge these disparities and improve maternal-fetal outcomes globally. A greater emphasis on individualized care plans, supported by evidence-based guidelines and emerging technologies, is essential for optimizing management of this high-risk population.

Maternal and fetal outcomes

The outcomes of pelvic fractures during pregnancy vary widely and depend largely on factors such as the mechanism and severity of trauma, the timing and appropriateness of medical intervention, and the availability of comprehensive multidisciplinary care. On the maternal side, outcomes span from full recovery to severe morbidity or even mortality. Hemorrhage remains the most serious and immediate life-threatening complication, occurring in roughly 30% of pelvic trauma cases during pregnancy, with the risk amplified by the increased vascularity of the pelvic region in late gestation [17]. In addition to hemorrhage, infection from open fractures or surgical intervention, thromboembolic events due to immobilization, and anesthesia-related complications can significantly impact maternal prognosis. A delay in diagnosis or suboptimal trauma care can further increase these risks, particularly in resource-limited settings.

Fetal outcomes are similarly complex and closely tied to maternal stability. Approximately 70% of fetuses survive maternal pelvic trauma; however, this statistic masks the high incidence of associated complications such as preterm labor (30-50%), placental abruption (20%), intrauterine growth restriction, and stillbirth (around 5%) [5,8,10,18,19]. Intrauterine growth restriction and preterm delivery, often medically indicated due to maternal instability or placental dysfunction, pose long-term developmental risks. Stillbirths most commonly result from placental detachment or direct uterine injury and are especially prevalent in high-energy trauma or when intervention is delayed.

The long-term consequences for mothers can be profound. Chronic pelvic pain, gait abnormalities, and persistent instability may impair physical function and quality of life. Furthermore, pelvic deformities or residual hardware may necessitate cesarean deliveries in future pregnancies and introduce complications such as malpresentation or obstructed labor [20]. Psychological sequelae, including post-traumatic stress disorder, anxiety, and depression, are frequently underreported but can be just as impactful as physical injuries.

For neonates, outcomes hinge on gestational age at delivery and the presence of trauma-related complications. Preterm infants, especially those born before 32 weeks, are at increased risk of respiratory distress, intraventricular hemorrhage, and long-term neurodevelopmental delays [18]. Low birth weight, a common consequence of intrauterine growth restriction or preterm labor, further compounds these risks and may necessitate prolonged neonatal intensive care.

Despite a growing body of literature on maternal-fetal trauma, there remains a disproportionate focus on acute outcomes, while long-term health implications are underexplored. The need for longitudinal cohort studies is critical to assess the full spectrum of maternal and neonatal sequelae, including reproductive challenges, functional limitations, and child development trajectories. Additionally, few management protocols incorporate formal mental health assessments or structured psychological support for mothers post-injury. Addressing these gaps through integrated, multidisciplinary postpartum care, including orthopedic rehabilitation and mental health services, could dramatically improve the quality of life for affected families. Finally, enhancing trauma registries to capture pregnancy-specific data could inform more targeted, evidence-based interventions.

Ethical and medico-legal considerations

The management of pelvic fractures during pregnancy is not only a clinical challenge but also an ethical and legal conundrum, requiring clinicians to carefully weigh competing obligations to both mother and fetus. Central to this complexity is the principle of maternal autonomy, which gives the pregnant woman the right to accept or refuse medical interventions. However, this autonomy can come into conflict with the perceived duty to protect fetal life, particularly in emergency scenarios where maternal decisions may endanger fetal viability. For instance, if a pregnant patient declines surgical intervention for religious or personal reasons, and that decision is likely to result in fetal demise or severe compromise, ethical tensions can become acute. Informed consent, therefore, must be approached with thoroughness and sensitivity, ensuring that the patient fully understands the potential implications for both herself and her unborn child [19].

From a legal perspective, the duty of care in obstetric trauma is dual-pronged, directed toward both the mother and fetus. Jurisdictions vary in how they prioritize these interests, and legal precedents often hinge on the gestational age of the fetus and its viability. For example, interventions to save a fetus at 34 weeks may be legally and ethically justifiable, even if they involve maternal risk, whereas the same interventions at 20 weeks might not be warranted. This variability makes the involvement of hospital ethics committees and legal counsel essential in high-stakes cases. Ethical dilemmas are further complicated in cases of brain death in the mother, where debates persist about maintaining somatic support for fetal maturation. Multidisciplinary ethics consultations can be instrumental in navigating these scenarios, integrating legal standards, clinical priorities, and respect for the patient’s values and cultural context [20].

The growing recognition of ethical and medico-legal complexities in managing trauma during pregnancy has not yet translated into robust clinical guidelines. Many healthcare providers feel underprepared to navigate these challenges, particularly in fast-paced trauma settings where decisions must be made rapidly. There is a pressing need to develop standardized ethical frameworks, clinical algorithms, and training modules tailored to trauma care in pregnancy. These tools should incorporate decision trees for maternal-fetal conflict, documentation best practices, and protocols for involving ethics and legal teams. Additionally, integrating ethical reasoning into obstetric trauma simulations could enhance provider confidence and decision-making capacity in real-world scenarios. As maternal trauma becomes more prevalent in global health discourse, the legal and ethical infrastructure must evolve in parallel to protect both patients and providers.

Case studies

Case studies offer invaluable, real-world insights into the diverse presentations, management strategies, and outcomes associated with pelvic fractures in pregnancy. These clinical narratives underscore the importance of multidisciplinary care, timely diagnosis, and individualized treatment planning. Table 1 summarizes notable cases from the literature, highlighting key clinical decisions and outcomes that have shaped current understanding and practice.

**Table 1:** Summary of selected case studies on pelvic fractures in pregnancy.

Case	Clinical scenario	Management strategy	Maternal and fetal outcomes	Clinical insight
1	A 40-year-old woman with superior and inferior pubic rami fractures and anterior hip dislocation following a motor vehicle accident	Conservative treatment with multidisciplinary care	Full maternal recovery; successful vaginal delivery	Importance of early intervention and collaborative management [21]
2	A retrospective review of 101 cases of pelvic trauma during pregnancy	Varied (conservative and surgical)	Maternal mortality: 9%; fetal mortality: 35%	Highlights the severity of trauma and the need for aggressive resuscitation [22]
3	A 27-year-old with insufficiency fractures secondary to pregnancy-induced osteoporosis	MRI-based diagnosis; elective cesarean section	Excellent maternal and neonatal outcomes	Significance of identifying osteoporosis-related fragility fractures [23]
4	A 33-year-old with spinal-pelvic dissociation due to culture-negative extrapulmonary tuberculosis	Surgical stabilization	Both mother and fetus survived	Reinforces the value of early diagnosis and integrated care [24]
5	Two cases of osteogenesis imperfecta in pregnancy	Genetic counseling and planned cesarean delivery	Both pregnancies successfully managed	Emphasizes the role of genetics and close prenatal monitoring [25]
6	An unstable pelvic fracture with hemorrhagic shock	Emergency cesarean section and splenectomy	Maternal survival; successful fetal delivery	Demonstrates the effectiveness of multidisciplinary trauma response [26]
7	Four women with unstable pelvic fractures	Surgical stabilization	Three successful pregnancies	Highlights the need for tailored surgical planning and prenatal follow-up [26]

These cases collectively illustrate the complexity and variability of pelvic fractures in pregnancy. They underscore the critical role of prompt, coordinated interventions and provide a foundation for refining clinical guidelines and future research directions.

Discussion

Pelvic fractures during pregnancy represent a convergence of high-stakes trauma management and delicate obstetric care. These cases demand immediate, coordinated responses that prioritize both maternal and fetal survival. Despite significant advancements in imaging and surgical interventions, many healthcare systems remain ill-equipped to manage such rare and complex scenarios, particularly in low-resource settings. The challenge lies not only in executing precise clinical interventions but also in developing comprehensive care models that integrate trauma protocols with maternal-fetal considerations. Persistent gaps in research, inconsistent access to multidisciplinary expertise, and limited long-term outcome data hinder progress in improving care standards across diverse populations.

Multidisciplinary Care

Robust evidence supports the notion that maternal survival rates can reach as high as 95% when multidisciplinary teams, including orthopedic surgeons, obstetricians, anesthesiologists, trauma specialists, and neonatologists, collaborate effectively [12]. However, such coordinated care is often a luxury limited to tertiary medical centers. In many regions, particularly in low- and middle-income countries, systemic barriers such as underfunded health infrastructure, insufficient trauma systems, and limited obstetric resources contribute to higher rates of maternal and fetal mortality [6,19]. To bridge this gap, it is essential to establish regional trauma referral networks, train frontline providers in pregnancy-specific trauma protocols, and utilize telemedicine to connect remote areas with specialized centers. Multidisciplinary simulation training and standardized trauma drills could also improve readiness and reduce decision-making delays. Importantly, integrating maternal-fetal medicine expertise into trauma teams from the outset ensures that both aspects of patient care receive equal attention. Promoting a culture of collaborative, protocol-driven care across institutions could drastically reduce complications and improve both maternal and neonatal outcomes.

Diagnostic Innovations

Modern imaging technologies such as low-dose CT and MRI offer unparalleled diagnostic precision, enabling clinicians to identify fracture patterns, vascular injuries, and associated soft tissue damage with confidence [10,11]. Yet, their use in pregnancy remains inconsistent due to persistent concerns about fetal radiation exposure and limited access in certain healthcare settings. The availability of MRI in emergency departments is still variable, and CT utilization often depends on provider experience and institutional policies. Furthermore, logistical challenges, such as transferring hemodynamically unstable patients to imaging suites, can impede timely diagnosis. The development and deployment of portable ultrasound devices present a promising solution, especially in trauma settings. Although ultrasound has limited value in evaluating bony structures, it plays a critical role in fetal monitoring, detecting intra-abdominal bleeding, and identifying placental abruption. Emerging modalities such as handheld MRI and artificial intelligence (AI)-enhanced image interpretation may enhance diagnostic capabilities in the future. However, systematic research is needed to validate these tools for use in pregnant trauma patients. Creating standardized imaging protocols and guidelines based on gestational age and injury severity could further streamline care and reduce diagnostic delays while ensuring maternal and fetal safety.

Management Challenges

Managing pelvic fractures in pregnant patients involves complex clinical decisions influenced by gestational age, fracture stability, maternal hemodynamics, and fetal viability. While conservative management, using pelvic binders, bed rest, and analgesia, is appropriate for stable fractures, it poses inherent risks such as thromboembolism, pressure ulcers, and prolonged hospital stays [14,15]. Conversely, surgical intervention in unstable fractures often becomes unavoidable, yet data regarding its long-term impact on maternal health and future pregnancies remain scarce [16]. Anesthetic exposure, intraoperative positioning, and fetal monitoring during surgery require careful coordination to avoid compromising uteroplacental perfusion. Advances such as minimally invasive fixation techniques and computer-assisted navigation have improved surgical outcomes, but most fixation devices were not designed for the biomechanical shifts and ligamentous laxity present during pregnancy. There is an urgent need for biomechanically tailored implants and stabilization strategies that account for the altered pelvic anatomy and weight distribution of pregnancy [7]. Furthermore, access to timely surgical care remains uneven globally, with resource-limited settings often lacking trauma-trained obstetric teams or surgical infrastructure. Developing scalable, cost-effective management pathways that include early mobilization, thromboprophylaxis, and multidisciplinary follow-up will be essential to improving maternal and fetal outcomes in both high- and low-resource settings.

This discussion highlights several persistent gaps in the current understanding and management of pelvic fractures during pregnancy. Key barriers include diagnostic limitations, a lack of pregnancy-specific surgical innovations, and disparities in access to multidisciplinary care. Equally concerning is the shortage of data on long-term maternal and child outcomes, such as physical function, future reproductive health, and developmental trajectories. Addressing these gaps will require sustained research efforts and a global commitment to innovation.

Longitudinal studies that track maternal recovery and neonatal development are essential for informing care strategies. Advances in diagnostics, such as AI-assisted imaging and portable MRI, alongside biomechanically adaptive fixation devices, offer promise but require validation in pregnant populations. Global initiatives should prioritize equitable access to trauma care by supporting mobile obstetric trauma teams, international clinical guidelines, and scalable care models for resource-limited settings.

Finally, integrating mental health support and social services into trauma recovery can improve long-term quality of life for mothers and families. Postpartum follow-up plays a critical role in optimizing both orthopedic and obstetric outcomes. Regular assessments allow early identification of complications such as pelvic instability, chronic pain, or impaired mobility, while also ensuring recovery of reproductive function, psychosocial well-being, and future pregnancy planning. A cross-disciplinary, systems-based approach, rooted in collaboration across orthopedics, obstetrics, mental health, and public health, will be critical to achieving sustained improvements in outcomes for both mother and child.

## Conclusions

Pelvic fractures during pregnancy, though rare, present complex risks to both maternal and fetal health and require coordinated, multidisciplinary management. Despite improvements in diagnostic and surgical approaches, outcomes remain inconsistent, particularly in resource-limited settings; however, maternal survival rates can approach 95% with cohesive team-based care. To improve outcomes, future efforts must prioritize standardized imaging, pregnancy-specific surgical strategies, structured postpartum follow-up, and public health initiatives aimed at trauma prevention. Addressing global disparities, strengthening ethical preparedness, and fostering interdisciplinary collaboration and research will be critical to advancing equitable, high-quality care for pregnant trauma patients.
